# Identification of distinct phenotypes and improving prognosis using metabolic biomarkers in COVID-19 patients

**DOI:** 10.62675/2965-2774.20240028-en

**Published:** 2024-07-18

**Authors:** Andressa Santana, Gabriele da Silveira Prestes, Marinara Dagostin da Silva, Carolina Saibro Girardi, Lucas dos Santos Silva, José Cláudio Fonseca Moreira, Daniel Pens Gelain, Glauco Adrieno Westphal, Emil Kupek, Roger Walz, Felipe Dal-Pizzol, Cristiane Ritter

**Affiliations:** 1 Laboratory of Experimental Pathophysiology Posgraduate Program in Health Sciences Universidade do Extremo Sul Catarinense Criciúma SC Brazil Laboratory of Experimental Pathophysiology, Posgraduate Program in Health Sciences, Universidade do Extremo Sul Catarinense - Criciúma (SC), Brazil.; 2 Center for Oxidative Stress Studies Instituto de Ciências Básicas da Saúde Universidade Federal do Rio Grande do Sul Porto Alegre RS Brazil Department of Biochemistry, Center for Oxidative Stress Studies, Instituto de Ciências Básicas da Saúde, Universidade Federal do Rio Grande do Sul - Porto Alegre (RS), Brazil.; 3 Centro Hospitalar Unimed Joinville SC Brazil Centro Hospitalar Unimed - Joinville (SC), Brazil.; 4 Public Health Department Universidade Federal de Santa Catarina Florianópolis SC Brazil Public Health Department, Universidade Federal de Santa Catarina - Florianópolis (SC), Brazil.; 5 Department of Clinical Medicine Hospital Universitário Universidade Federal de Santa Catarina Florianópolis SC Brazil Center for Applied Neuroscience, Department of Clinical Medicine, Hospital Universitário, Universidade Federal de Santa Catarina - Florianópolis (SC), Brazil.; 6 Intensive Care Unit Hospital São José Criciúma SC Brazil Intensive Care Unit, Hospital São José - Criciúma (SC), Brazil.

**Keywords:** COVID-19, Coronavirus infections, Hidrocortisone, Phenotype, Metabolism

## Abstract

**Objective:**

To investigate the relationship between the levels of adipokines and other endocrine biomarkers and patient outcomes in hospitalized patients with COVID-19.

**Methods:**

In a prospective study that included 213 subjects with COVID-19 admitted to the intensive care unit, we measured the levels of cortisol, C-peptide, glucagon-like peptide-1, insulin, peptide YY, ghrelin, leptin, and resistin.; their contributions to patient clustering, disease severity, and predicting in-hospital mortality were analyzed.

**Results:**

Cortisol, resistin, leptin, insulin, and ghrelin levels significantly differed between severity groups, as defined by the World Health Organization severity scale. Additionally, lower ghrelin and higher cortisol levels were associated with mortality. Adding biomarkers to the clinical predictors of mortality significantly improved accuracy in determining prognosis. Phenotyping of subjects based on plasma biomarker levels yielded two different phenotypes that were associated with disease severity, but not mortality.

**Conclusion:**

As a single biomarker, only cortisol was independently associated with mortality; however, metabolic biomarkers could improve mortality prediction when added to clinical parameters. Metabolic biomarker phenotypes were differentially distributed according to COVID-19 severity but were not associated with mortality.

## INTRODUCTION

Different clinical phenotypes have been attributed to coronavirus disease 2019 (COVID-19) pneumonia; some patients have mild to moderate respiratory symptoms, and others require hospitalization and mechanical ventilation.^(1)^ Patients with poor clinical outcomes include those with metabolic disorders such as obesity and diabetes.^(1-3)^ These findings suggest that dysfunctional adipose tissue and metabolism play a role in regulating systemic and pulmonary inflammatory responses against severe acute respiratory syndrome coronavirus 2 (SARS-CoV-2), leading to excessive lung injury and respiratory failure.^(4)^

A convergence point for this could be insulin resistance.^(5)^ It has been suggested that angiotensin-converting enzyme 2 (ACE2) is an important molecular link between insulin resistance and COVID-19 severity.^(6,7)^ Insulin resistance is also associated with a chronic proinflammatory state, which can induce a more severe cytokine storm during COVID-19.^(8)^ Additionally, adipokines, such as ghrelin, leptin, and resistin, have been extensively studied in many chronic disorders and are widely related to obesity and insulin resistance; however, their precise role in critical illness is largely unknown.^(9)^ Ghrelin, leptin, and resistin have been associated with inflammatory host responses in critically ill patients.^(10)^ Leptin, for instance, is implicated in cell-mediated immunity and cytokine crosstalk, and hyperresistinemia might be fundamental in persistent inflammation that occurs in septic shock.^(11)^ It has been hypothesized that adipokines are the missing link between obesity and outcomes in patients with severe COVID-19, but no robust evidence to support this hypothesis has been reported to date.^(12,13)^Recently, it was demonstrated that circulating adipokine levels are associated with COVID-19 hospitalization or the need for mechanical ventilation but not with mortality.^(14)^

Other endocrine biomarkers (EBs), such as cortisol, C-peptide, glucagon-like peptide-1 (GLP-1), insulin, and peptide YY (PYY), appear to be associated with clinical outcomes in hospitalized patients with COVID-19.^(15-19)^ Serum cortisol levels appear to be an independent predictor of unfavorable outcomes, and there is an apparent association between other metabolic markers and inflammation modulatory pathways in patients with COVID-19.^(15-19)^

Patients who are overweight, obese, or have metabolic disorders tend to have different serum levels of adipokines and EB (cortisol, C-peptide, GLP-1, insulin, and PYY) compared to those without metabolic comorbidities, which could be associated with poor outcomes in patients with COVID-19. Therefore, the aim of this prospective cohort study was to investigate the relationships between the levels of adipokines and other EBs (collectively referred to as metabolic biomarkers, MBs) and outcomes in hospitalized patients with COVID-19 to establish prognostic clusters based on clinical and laboratory parameters.

## METHODS

### Study design

A prospective cohort study was conducted on subjects admitted to six intensive care units (ICUs) in two tertiary hospitals in southern Brazil between June and November 2020. The study was performed in accordance with the Declaration of Helsinki and the Brazilian National Health Council Resolution 466. The Ethics Committee of *Hospital São José* (31384620.6.1001.5364) and *Centro Hospitalar Unimed* (31384620.6.2002.5362) approved the protocol. All subjects or their surrogates provided written informed consent before inclusion in the study.

### Settings

The study sample consisted of consecutive subjects admitted to the ICUs of the participating hospitals from June to November 2020.

### Participants

Subjects aged older than 18 who were diagnosed with COVID-19 through reverse-transcription polymerase chain reaction or a rapid antigen test and who required supplemental oxygen (World Health Organization [WHO] class 5), noninvasive ventilation (WHO class 6), or invasive mechanical ventilation (WHO class 7) due to COVID-19 pneumonia were included in the study.^(20)^ Subjects who were asymptomatic (WHO class 1) or symptomatic (WHO class 2, ambulatory mild disease, independent) were included as a control group. The control group was selected by convenience advertising the study on e-mail lists from the university and hospital participating in the study. Subjects with severe chronic diseases (e.g., chronic kidney disease resulting from dialysis, Child‒Pugh class C cirrhosis, severe chronic obstructive pulmonary disease, severe heart failure) or diseases that alter the inflammatory response, such as those requiring long-term use of immunosuppressants, active cancer, poorly controlled human immunodeficiency virus infection, and receiving palliative care or with a life expectancy of less than 24 hours, as judged by the attending physician, were excluded.

### Procedures

Following enrollment, venous blood samples were collected within 24 hours after ICU admission. It was not possible to ascertain whether blood was collected before or after corticosteroid administration. Sociodemographic and clinical information was collected directly from the patients or their surrogate or electronic medical records. The levels of MB (cortisol, C-peptide, GLP-1, insulin, PYY, ghrelin, leptin, and resistin) were measured using the Metabolism/Obesity 9-Plex Human ProcartaPlex^TM^ Panel 2 (Cat. #EPX060-10824-901, Thermo Fisher Scientific Waltham, Massachussets, USA) and a Luminex® MAGPIX system (Luminex Corporation, Austin, Texas, USA). Protein concentrations were calculated using the online Procarta Plex Analysis Application (Thermo Fisher Scientific).

### Statistical analysis

Continuous variables are expressed as the mean and standard deviation (SD). Categorical data are expressed as frequencies and percentages. Survivor and Nonsurvivor groups were compared using Kruskal‒Wallis and Wilcoxon‒Mann‒Whitney tests for continuous outcomes and chi‒square tests for categorical outcomes, and multivariable binary logistic regression was used to analyze the adjusted differences between groups. Additionally, multivariable binary logistic regression was used to determine the associations between the primary outcome, hospital mortality, and independent variables, which were conceptually divided into core demographic, including age and sex, and clinical prognostic factors (Simplified Acute Physiology Score [SAPS] III, Charlson Comorbidity Index, chest computed tomography [CT] score, Sequential Organ Failure Assessment [SOFA] and body mass index [BMI]), hereafter referred to as “core predictors,” and the MB. The latter were categorized into quintiles owing to their highly nonnormal distributions. Variables were entered into the regression if a threshold of p < 0.20 was reached in the univariate analysis or if there was biological plausibility associated with the outcome. The receiver operating characteristic (ROC) curves for the core predictors alone and the core predictors plus the MB predictors were compared in terms of accuracy, measured using the area under the ROC curve (AUROC), as well as the sensitivity, specificity, positive and negative predictive values, and likelihood ratios of positive and negative tests. The AUROC was cross-validated on five independent samples to avoid model fitting and evaluation of the same sample. Bootstrap bias-corrected 95% confidence intervals (95%CIs) were used to express AUROC uncertainty. Spearman’s correlation coefficient was used to assess pairwise correlations between the numerical variables. A hierarchical cluster of the corresponding correlation matrix was used to identify the clusters of coexpressed biomarkers. Additionally, a nonhierarchical *K*-means clustering analysis was performed to assess MB clustering.

The data were analyzed using IBM SPSS Statistics version 22.0 (IBM Corp., Armonk, New York, USA) and Stata version 13.1 (StataCorp, College Station, Texas, USA). The type I error level was set to 0.05 for all the statistical analyses.

## RESULTS

### Participant characteristics

Two hundred thirteen subjects were included in this study, of whom 53 (25%) died during hospitalization; a comparison between those who died and those who survived is shown in table 1. Older age, higher BMI, need for mechanical ventilation, extension of COVID-19 pneumonia on CT, disease severity on ICU admission (measured using the SAPS III), comorbidities (measured using the Charlson Comorbidity Index), and degree of organ dysfunction (measured using the SOFA) were associated with in-hospital mortality. After adjustment, only the SOFA score at admission and the SAPS III score were independently associated with in-hospital mortality.

**Table 1:** Participant characteristics

	Survivor (n = 160)	Nonsurvivor (n = 53)	Unadjusted p value	Adjusted OR (95%CI)
Sex, male	99 (62)	37 (70)	0.29	NA
Age	53 ± 15	62 ± 13	< 0.001	1.02 (0.98 - 1.07)
Ethnicity, white	152 (95)	48 (90)	0.24	NA
BMI	26 ± 10	29 ± 5	0.026	1.02 (0.95 - 1.09)
Time from the beginning of symptoms (days)	10.5 ± 3.7	10.9 ± 4.2	0.67	NA
Transferred from				
Emergency Department	115 (72)	37 (70)		NA
COVID-19 ward	9 (6)	4 (8)	0.87	
Other hospital	36 (22)	12 (22)		
Mechanical ventilation need	46 (29)	37 (70)	< 0.001	NA
Thorax CT scan extension of lesions > 50%	51 (32)	28 (53)	0.006	0.87 (0.5 - 1.5)
Charlson comorbidity index	1.62 ± 1.5	2.34 ± 1.65	0.004	0.94 (0.67 - 1.3)
Diabetes mellitus	14 (9)	7 (13)	0.34	NA
Corticosteroid use	122 (76)	44 (83)	0.30	NA
SOFA at admission	2 [1 - 4]	4 [2 - 8]	< 0.001	1.2 (1.09 - 1.5)
SAPS III score	39 ± 25	58 ± 20	< 0.001	1.02 (1.004 - 1.04)

OR - odds ratio; 95%CI - 95% confidence interval; NA - not applied; BMI - body mass index; CT - computed tomography; SOFA - Sequential Organ Failure Assessment; SAPS - Simplified Acute Physiologic Score. The results are expressed as the n (%), mean ± standard deviation or median [interquartile range].

### Metabolic biomarkers and disease severity

**Figure 1:**
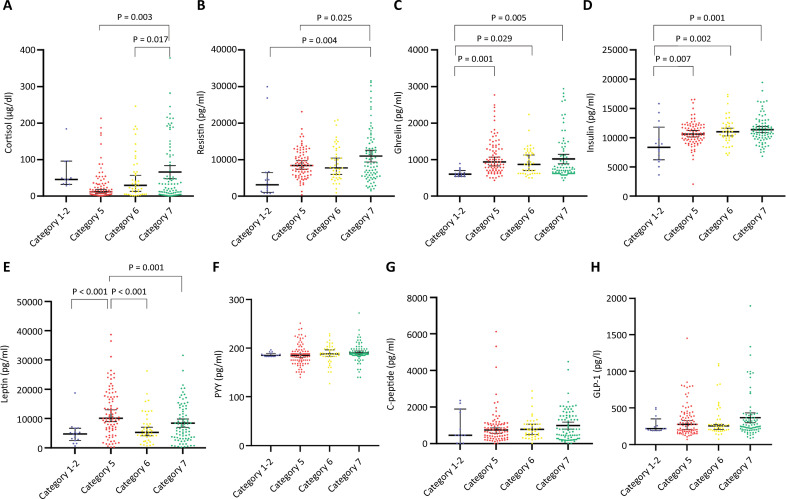
Metabolic biomarkers and COVID-19 severity.

**Figure 2:**
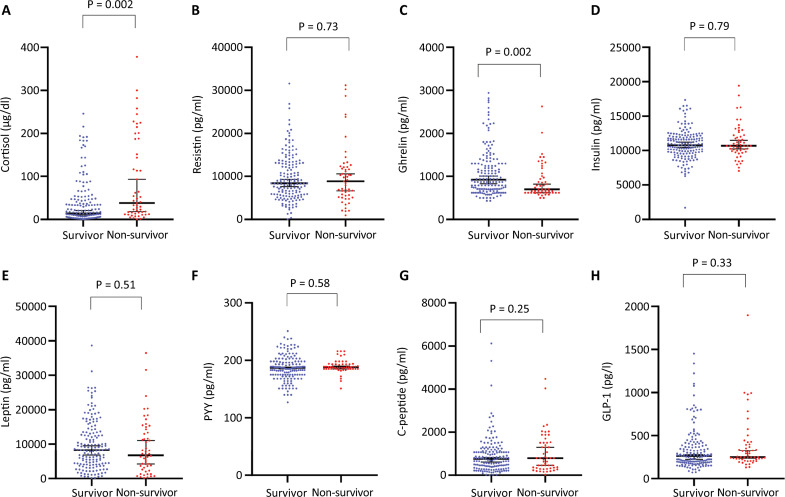
Metabolic biomarkers and COVID-19 mortality. PYY - peptide YY; glucagon-like peptide-1.

**Figure 3:**
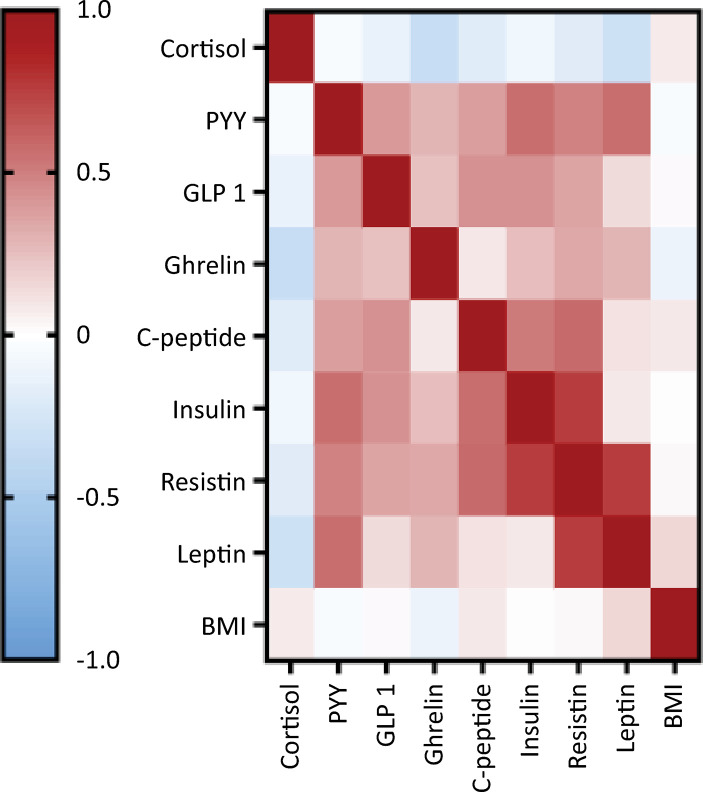
Spearman’s correlations between metabolic biomarker levels and body mass index. PYY - peptide YY; GLP-1 - glucagon-like peptide 1; BMI - body mass index.

In the analysis of participants according to the WHO severity scale, cortisol, resistin, leptin, insulin, and ghrelin levels significantly differed between groups (Figure 1). Plasma cortisol and resistin levels were greater in mechanically ventilated subjects than in those with less severe disease (Figure 1A and B). Ghrelin and insulin levels were greater in hospitalized subjects (WHO categories 5 - 7) than in those with mild disease (Figure 1C and D). Additionally, leptin levels were greater in subjects with WHO category 5 disease than in subjects with WHO categories 2 - 3 and 6 or 7 disease (Figure 1E). Lower ghrelin and higher cortisol levels were the only variables significantly associated with mortality (Figure 2); however, after adjusting for clinical variables, a higher cortisol level was the only biomarker independently associated with mortality (odds ratio [OR]: 1.003; 95%CI 1.00 - 1.006; AUROC 0.61 [95%CI] 0.51 - 0.7). Body mass index and measured MB were not significantly correlated, except for a weak correlation with leptin level (r = 0.15, p = 0.028) (Figure 3). Since cortisol could have a nonlinear association with mortality, we analyzed quartiles of cortisol levels, but only higher cortisol levels were associated with mortality (Table 2).

**Table 2:** Quartiles of cortisol levels and mortality

Cortisol levels	Survivor (n = 160)	Nonsurvivor (n = 53)	p value
25 quartile	46 (82)	10 (18)	
25 - 50 quartile	47 (84)	9 (16)	0.046
50 - 75 quartile	34 (65)	18 (35)	
75 quartile	33 (67)	16 (33)	

The results are expressed as the n (%).

**Figure 4:**
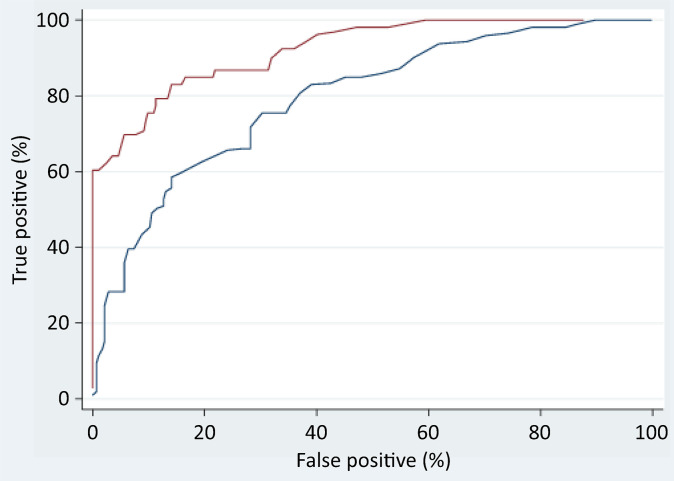
ROC curve comparison between the ability of clinical (blue) and clinical plus biomarker (brown) variables to predict in-hospital mortality among COVID-19 patients, as determined by multivariate logistic regression and 5-fold cross-validation.

Multivariate logistic regression was used to investigate the ability of MBs to predict in-hospital mortality. Adding MB to the core clinical predictors (Table 3) significantly improved the AUROC from 90.31% (95%CI: 70.78 - 85.33%) to 92.65% (95%CI: 86.86 -95.74%). Significant improvements were also observed for sensitivity (from 40 to 70%, p < 0.001), negative predictive value (from 82 to 89%, p = 0.014), accuracy (from 80 to 93%, p = 0.003), and the likelihood ratios of a positive/negative test (p < 0.001). Doubling the likelihood ratio of a positive test with the addition of MB doubled the odds of correctly predicting in-hospital mortality. Within the range of 10 - 15% false-positive test results, which is considered acceptable in many screening applications, the MB improved the correct prediction of in-hospital mortality by approximately 10 - 30% compared with the ROC without the MB (Table 3 and Figure 4).

**Table 3:** Diagnostic parameter comparison of two in-hospital survival models derived by multivariable logistic regression and 5-fold cross-validation

Parameter name	Clinical predictors			Clinical + MB predictors			Parameter difference p value
	Value	95%CI bounds		Value	95%CI bounds		
		Lower	Upper		Lower	Upper	
Sensitivity	39.6	27.59	53.06	69.81	56.46	80.48	< 0.001
Specificity	94.3	89.28	97.12	95.07	90.17	97.59	0.400
PPV	72.4	54.58	85.30	84.09	71.00	92.00	0.112
NPV	81.7	74.05	86.00	89.40	83.48	93.47	0.014
AUROC	80.4	70.78	85.33	92.65	86.86	95.74	0.003
LRT+	7.03	4.77	10.36	14.16	10.46	19.17	< 0.001
LRT-	0.64	0.60	0.68	0.32	0.28	0.36	< 0.001

MB - metabolic biomarker; 95%CI - 95% confidence interval; PPV - positive predictive value; NPV - negative predictive value; AUROC - area under the receiver operating curve; LRT+ - likelihood ratio of a positive test; LRT- - likelihood ratio of a negative test; The results are expressed as the %. The results are expressed as the n (%), mean ± standard deviation.

### Clustering based on metabolic biomarkers

**Figure 5:**
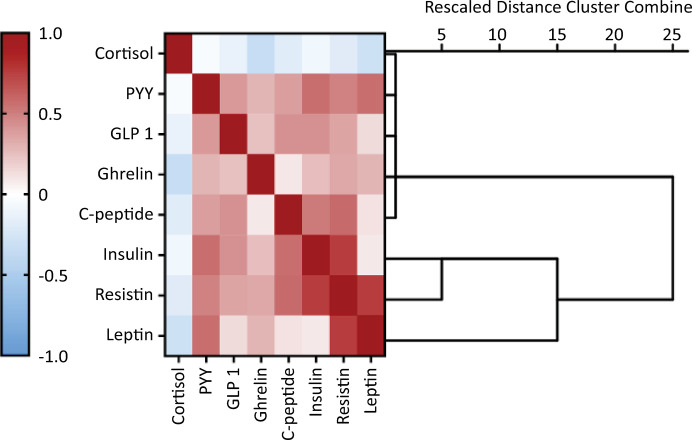
Spearman´s correlations between metabolic biomarker levels and hierarchical cluster analysis. PYY - peptide YY; GLP-1 - glucagon-like peptide-1. The magnitude of each correlation is denoted with a color, where the red color indicates a positive correlation and the blue color indicates a negative correlation.

Spearman’s correlation coefficient was used to assess the pairwise correlation of the measured biomarkers, and the clusters of hierarchical coexpressed biomarkers are shown in the corresponding correlation matrix (Figure 5). The cluster between insulin/resistin and leptin reinforced the observed association between these biomarkers and disease severity (Figure 1). This hierarchical cluster was extended to ghrelin in the next step (Figure 5), and ghrelin was associated with disease severity. Notably, cortisol, which is associated with disease severity and is the only biomarker independently associated with mortality, did not cluster with these biomarkers (Figure 5). To further explore this potential relationship, a nonhierarchical *K*-means cluster analysis was performed. Using all the measured biomarkers, two phenotypes were identified: phenotype 1, which included 147 subjects, and phenotype 2, which included 66 subjects. The demographic and clinical characteristics were similar between the two phenotypes (Table 4). The distinct phenotypes were associated with the WHO severity scale but not mortality (Table 4). Similar results were obtained using the nonhierarchical *K*-means cluster to guide the correlation matrix and hierarchical cluster to select a few biomarkers to determine the phenotypes. Similar phenotyping was possible using only leptin and insulin levels.

**Table 4:** Characteristics of biomarker clusters

	Cluster 1 (n = 147)	Cluster 2 (n = 66)	p value
Gender, male	98 (67)	38 (58)	0.20
Age	56 ± 16	53 ± 14	0.17
BMI	27 ± 9	28 ± 9	0.23
Time from the beginning of symptoms (days)	10.7 ± 4.1	10.5 ± 3.6	0.91
Transferred from			
Emergency Department	108 (73)	44 (67)	0.40
COVID-19 ward	7 (5)	6 (9)	
Other hospital	32 (22)	16 (24)	
Thorax CT scan extension of lesions > 50%	58 (39)	21 (32)	0.48
Charlson comorbidity index	1.9 ± 1.5	1.5 ± 1.5	0.12
Diabetes mellitus	17 (12)	4 (6)	0.21
Corticosteroid use	115 (78)	51 (77)	0.87
SAPS III score	42 ± 26	50 ± 23	0.13
SOFA at admission	3.6 ± 2.9	3.0 ± 2.7	0.081
Cortisol levels	41 ± 63	18 ± 37	0.002
C-peptide levels	839 ± 858	1258 ± 1407	0.002
GLP levels	310 ± 255	424 ± 255	< 0.001
Ghrelin levels	957 ± 483	1199 ± 564	< 0.001
Insulin levels	10845 ± 2642	11882 ± 2682	0.001
Leptin levels	5581 ± 3442	18390 ± 5994	< 0.001
PYY levels	188 ± 25	189 ± 18	0.627
Resistin levels	8654 ± 5338	12229 ± 6111	< 0.001
WHO severity scale			
Category 5	53 (36)	34 (51)	0.03
Category 6	36 (24)	7 (11)	
Category 7	58 (40)	25 (38)	
Nonsurvivor	36 (24)	17 (26)	0.84

BMI - body mass index; CT - computed tomography; SAPS - Simplified Acute Physiologic Score; SOFA - Sequential Organ Failure Assessment; GLP - glucagon-like peptide; PYY - peptide YY; WHO - World Health Organization; NA - not applied. The results are expressed as the n (%), mean ± standard deviation

## DISCUSSION

In this multicenter study examining a large cohort of patients with COVID-19, we found that different MBs and patient phenotypes based on MBs were associated with disease severity, but only cortisol levels were independently associated with mortality. Additionally, MB improved the ability of the clinical variables to predict mortality. It is not possible to ascertain whether these biomarkers are significantly involved in the progression of the disease to more severe forms or if they are simply an epiphenomenon of the complex pathological process that occurs during COVID-19 development.

A notable finding of this study is that we did not find any significant correlation between BMI and measured MB, except for leptin levels. Even for leptin, the correlation was weak despite its statistical significance. This finding is different from what has been observed in healthy individuals^(21)^ but is similar to the findings of another recent study.^(14)^ Flikweert et al.^(14)^ hypothesized that COVID-19 can induce adipose tissue dysfunction and altered the secretion of adipokines, but this pattern has been observed in patients with sepsis,^(22)^ suggesting that a complex interplay exists between adipose tissue and inflammation in these conditions; additionally, this could be secondary to the lower mean BMI observed in our cohort, where only 39% (84 out of 213 patients with a BMI ≥ 30) were overweight.

Cortisol is the most studied biomarker to date. In this study, higher cortisol levels were associated with in-hospital mortality and appeared to be an independent prognostic marker in critically ill patients with COVID-19. Cortisol levels are relatively well established in the literature as a severity marker and mortality predictor in patients with COVID-19.^(23-27)^

There is a paucity of large cohort studies on MB other than cortisol and COVID-19 severity. Ghrelin receptors have been detected in lung tissue, and studies have shown that ghrelin has protective effects on acute lung injury models.^(28)^ In this study, higher ghrelin levels were found in COVID-19 survivors. Ghrelin levels are inversely correlated with the length of mechanical ventilation and ICU stay, and higher serum ghrelin levels are correlated with survival in patients with sepsis.^(29)^ However, we did not find any prospective studies determining the role of ghrelin in predicting the prognosis of patients with COVID-19.

In patients with COVID-19, leptin modulation of the inflammatory system can directly contribute to the pathophysiology of the disease because high leptin levels are associated with a proinflammatory response and infection severity in obese and nonobese patients.^(30,31)^ Leptin appears to be protective against respiratory infections, and serum leptin levels are inversely proportional to inflammatory biomarkers in patients hospitalized for pneumonia.^(32)^ In contrast, during viral infections in obese patients, altered leptin levels can lead to a proinflammatory cytokine response and a deficient response to infection.^(33)^ Patients with COVID-19 generally have higher serum leptin levels than healthy controls, but the relationship between serum leptin levels and COVID-19 severity is not robust.^(14,34-38)^ Our results reflect this uncertainty. Leptin levels were greater in WHO category 5 than in all other categories but were not associated with mortality.

Resistin levels are increased in patients with sepsis and are associated with mortality independent of BMI in patients with sepsis.^(39,40)^Resistin levels are greater in patients with COVID-19 than in healthy controls,^(41)^ and resistin is correlated with disease severity and outcome, irrespective of the presence of obesity or metabolic syndrome.^(14, 42,43)^Our results are consistent with these previous findings: resistin levels were greater in patients with WHO category 7 disease, independent of BMI, but did not predict mortality.

We also studied C-peptide, GLP-1, insulin, and PYY, but insulin levels were the only EBs that were greater in patients with more severe forms of the disease than in patients with WHO category 1 and 2 diseases. To the best of our knowledge, none of these markers have been studied as prognostic biomarkers for COVID-19. As observed in sepsis, COVID-19 can induce hypermetabolic stress, which is associated with hyperglycemia and insulin resistance.^(44)^ Hyperinsulinism is an adaptation response to the excessive release of counterregulatory hormones, and this could partially explain our results.

Independent of clinical parameters, patients with COVID-19 could be clustered into two phenotypes associated with disease severity. This approach could be useful for delivering more personalized care. In another study, five phenotypes of hospitalized COVID-19 patients were identified using creatinine, albumin, CRP, white blood cell count, and clinical characteristics.^(45)^ Patients with a phenotype characterized by renal failure, hypoalbuminemia, anemia, lymphopenia, and elevated CRP levels had the highest likelihood of ICU transfer or in-hospital mortality. Three subphenotypes, namely, a history of chronic hypertension, fever, respiratory and nonrespiratory symptoms, and age, have been found to be associated with clinical deterioration.^(46)^ Additionally, some phenotypes were found to be associated with better outcomes after the introduction of dexamethasone therapy for treating COVID-19, reinforcing the idea that phenotyping patients can have an impact on prognosis and treatment stratification.^(47)^ To the best of our knowledge, this study is the first to provide discriminative phenotypes based only on EB levels.

Few prospective studies have included a large number of patients from different centers, which is a major strength of this study. Additionally, this study is the first to show that MBs can improve the prognostic accuracy of clinical parameters and can be used to phenotype patients independent of clinical parameters. However, this study also has several limitations. First, MBs were analyzed at a single time point after hospital admission, leading to a lack of information on metabolic modifications over time and their association with disease progression. This is one of the great challenges of biomarker use and phenotyping in complex diseases such as COVID-19. We believe that this limitation could be mitigated by the fact that biomarkers add to the prognostic value of relevant clinical parameters. Additionally, since the time elapsed between the self-reported beginning of COVID-19 symptoms and study inclusion was similar between groups, this could reduce the possible temporal bias related to a single time point measurement of biomarkers. Second, some relevant adipokines, such as adiponectin and visfatin, were not measured, thus preventing a comprehensive assessment of the role of MBs in patients with COVID-19. Third, glycemic control during the ICU could affect mortality and be impacted by dexamethasone use, but unfortunately, we do not have information on these parameters (such as median capillary blood glucose and glycosylated hemoglobin), which should be noted as a limitation of our study. However, some studies have not associated glycemic control with disease severity even in diabetic patients receiving dexamethasone.^(48)^ Fifth, MB phenotypes could also impact long-term outcomes, since persistent muscle atrophy, new onset of diabetes, and other metabolic outcomes are associated with COVID-19,^(49-51)^and further studies are required to assess the role of MB in predicting the long-term outcomes of patients with COVID-19. Sixth, we only have information on corticosteroid use, not the dose of the drug administered; thus, although the number of patients who used corticosteroids was similar among survivors and nonsurvivors, it is possible to have some confounders, mainly related to the dose used. However, the institutional protocol suggested the use of dexamethasone 6mg, which could partially attenuate this bias. Seventh, during the data analysis, we performed multiple comparisons, and this intrinsically carries a potential bias, which must be taken into account when reading our results.

## CONCLUSION

Cortisol was the only single biomarker independently associated with mortality; however, metabolic biomarkers improved mortality prediction when added to clinical parameters. Metabolic biomarker phenotypes were differentially distributed according to COVID-19 severity but were not associated with mortality. Taken together, these results suggest that MB may play a role in COVID-19 progression and could reflect an imbalance in different pathways affected by SARS-CoV-2 infection.
