# Supplementary material for: Identification of distinct phenotypes and improving prognosis using metabolic biomarkers in COVID-19 patients
Source: Crit Care Sci. 2024 Jul 18;36:e20240028en. doi: 10.62675/2965-2774.20240028-en (PMC11321718; doi:10.62675/2965-2774.20240028-en)
Supplement: Translation

# Translation: Identificação de fenótipos distintos e melhoria do prognóstico usando biomarcadores metabólicos em pacientes com COVID-19

**DOI:** 10.62675/2965-2774.20240028-pt

**Authors:** Andressa Santana, Gabriele da Silveira Prestes, Marinara Dagostin da Silva, Carolina Saibro Girardi, Lucas dos Santos Silva, José Cláudio Fonseca Moreira, Daniel Pens Gelain, Glauco Adrieno Westphal, Emil Kupek, Roger Walz, Felipe Dal-Pizzol, Cristiane Ritter

**Affiliations:** 1 Brasil Laboratório de Fisiopatologia Experimental, Programa de Pós-Graduação em Ciências da Saúde, Universidade do Extremo Sul Catarinense - Criciúma (SC), Brasil.; 2 Brasil Departamento de Bioquímica, Centro de Estudos de Estresse Oxidativo, Instituto de Ciências Básicas da Saúde, Universidade Federal do Rio Grande do Sul - Porto Alegre (RS), Brasil.; 3 Brasil Centro Hospitalar Unimed - Joinville (SC), Brasil.; 4 Brasil Departamento de Saúde Pública, Universidade Federal de Santa Catarina - Florianópolis (SC), Brasil.; 5 Brasil Centro de Neurociência Aplicada, Departamento de Medicina Clínica, Hospital Universitário, Universidade Federal de Santa Catarina - Florianópolis (SC), Brasil.; 6 Brasil Unidade de Terapia Intensiva, Hospital São José - Criciúma (SC), Brasil.

**Keywords:** COVID-19, Infecções por coronavírus, Hidrocortisona, Fenótipo, Metabolismo

## Abstract

**Objetivo:**

Investigar a relação entre os níveis de adipocinas e outros biomarcadores endócrinos e os desfechos em pacientes hospitalizados com COVID-19.

**Métodos:**

Estudo prospectivo que incluiu 213 indivíduos com COVID-19 internados na unidade de terapia intensiva, em que foram medidos os níveis de cortisol, peptídeo-C, peptídeo-1 semelhante ao glucagon, insulina, peptídeo YY, grelina, leptina e resistina; analisamos suas contribuições para o agrupamento de pacientes, gravidade da doença e previsão de mortalidade hospitalar.

**Resultados:**

Os níveis de cortisol, resistina, leptina, insulina e grelina diferiram significativamente entre os grupos por gravidade, conforme definido pela escala de gravidade da Organização Mundial da Saúde. Além disso, níveis mais baixos de grelina e mais altos de cortisol foram associados à mortalidade. A adição de biomarcadores aos preditores clínicos de mortalidade melhorou significativamente a precisão na determinação do prognóstico. A fenotipagem de indivíduos com base nos níveis de biomarcadores plasmáticos produziu dois fenótipos diferentes, que foram associados à gravidade da doença, mas não à mortalidade.

**Conclusão:**

Como único biomarcador, apenas o cortisol foi independentemente associado à mortalidade; no entanto, os biomarcadores metabólicos podem melhorar a previsão de mortalidade quando associados a parâmetros clínicos. Os fenótipos dos biomarcadores metabólicos foram distribuídos diferencialmente, conforme a gravidade da COVID-19, mas não foram associados à mortalidade.

## INTRODUÇÃO

Diferentes fenótipos clínicos foram atribuídos à pneumonia causada pela doença do coronavírus 2019 (COVID-19). Alguns pacientes apresentam sintomas respiratórios leves a moderados, e outros necessitam de hospitalização e ventilação mecânica.^(1)^ Os pacientes com desfechos clínicos desfavoráveis incluem aqueles com distúrbios metabólicos, como obesidade e diabetes.^(1-3)^ Esses achados sugerem que o tecido adiposo e o metabolismo disfuncionais desempenham papel na regulação das respostas inflamatórias sistêmicas e pulmonares contra o coronavírus da síndrome respiratória aguda grave 2 (SARS-CoV-2), levando a lesão pulmonar excessiva e à insuficiência respiratória.^(4)^

Um ponto de convergência para isso pode ser a resistência à insulina.^(5)^ Sugeriu-se que a enzima conversora de angiotensina 2 (ECA2) é um importante elo molecular entre a resistência à insulina e a gravidade da COVID-19.^(6,7)^ A resistência à insulina também está associada a um estado pró-inflamatório crônico, que pode induzir uma tempestade de citocinas mais grave durante a COVID-19.^(8)^ Além disso, as adipocinas, como a grelina, a leptina e a resistina, foram amplamente estudadas em muitos distúrbios crônicos e estão amplamente relacionadas à obesidade e à resistência à insulina; no entanto, seu papel preciso na doença crítica é amplamente desconhecido.^(9)^ A grelina, a leptina e a resistina têm sido associadas a respostas inflamatórias do hospedeiro em pacientes em estado crítico.^(10)^ A leptina, por exemplo, está implicada na imunidade mediada por células e na interação de citocinas, e a hiperresistinemia pode ser fundamental na inflamação persistente que ocorre no choque séptico.^(11)^ Cogitou-se a hipótese de que as adipocinas são o elo perdido entre a obesidade e os resultados em pacientes com COVID-19 grave, mas, até o momento, não foi relatada evidência robusta para apoiar essa hipótese.^(12,13)^ Recentemente, foi demonstrado que os níveis de adipocinas circulantes estão associados à hospitalização por COVID-19 ou à necessidade de ventilação mecânica, mas não à mortalidade.^(14)^

Outros biomarcadores endócrinos (BEs), como o cortisol, o peptídeo-C, o peptídeo-1 semelhante ao glucagon (GLP-1), a insulina e o peptídeo YY (PYY), parecem estar associados a desfechos clínicos em pacientes hospitalizados com COVID-19.^(15-19)^ Os níveis séricos de cortisol parecem ser um preditor independente de desfechos desfavoráveis, e há uma aparente associação entre outros marcadores metabólicos e vias moduladoras da inflamação em pacientes com COVID-19.^(15-19)^

Pacientes com sobrepeso, obesidade ou distúrbios metabólicos tendem a ter níveis séricos diferentes de adipocinas e BEs (cortisol, peptídeo-C, GLP-1, insulina e PYY) em comparação com aqueles sem comorbidades metabólicas, o que poderia estar associado a desfechos desfavoráveis em pacientes com COVID-19. Assim, o objetivo deste estudo de coorte prospectivo foi investigar as relações entre os níveis de adipocinas e outros BEs (coletivamente chamados de biomarcadores metabólicos, BMs) e os desfechos em pacientes hospitalizados com COVID-19, para estabelecer agrupamentos de prognóstico a partir de parâmetros clínicos e laboratoriais.

## MÉTODOS

### Desenho do estudo

Trata-se de estudo de coorte prospectivo realizado em indivíduos admitidos em seis unidades de terapia intensiva (UTIs) em dois hospitais terciários no Sul do Brasil entre junho e novembro de 2020. O estudo foi realizado de acordo com a Declaração de Helsinque e a Resolução 466 do Conselho Nacional de Saúde do Brasil. Os Comitês de Ética do Hospital São José (31384620.6.1001.5364) e do Centro Hospitalar Unimed (31384620.6.2002.5362) aprovaram o protocolo. Todos os indivíduos ou seus substitutos forneceram consentimento livre e esclarecido por escrito antes da inclusão no estudo.

### Contexto

A amostra do estudo consistiu em indivíduos consecutivos admitidos nas UTIs dos hospitais participantes de junho a novembro de 2020.

### Participantes

Foram incluídos no estudo indivíduos com mais de 18 anos de idade que foram diagnosticados com COVID-19 por meio da reação em cadeia da polimerase com transcrição reversa ou de teste rápido de antígeno e que necessitaram de oxigênio suplementar (classe 5 da Organização Mundial da Saúde [OMS]), ventilação não invasiva (classe 6 da OMS) ou ventilação mecânica invasiva (classe 7 da OMS) devido à pneumonia por COVID-19.^(20)^ Os indivíduos assintomáticos (classe 1 da OMS) ou sintomáticos (classe 2 da OMS, doença leve ambulatorial, independente) foram incluídos como grupo controle. O grupo controle foi selecionado por conveniência, anunciando o estudo em listas de *e-mail* da universidade e do hospital que participaram do estudo. Foram excluídos indivíduos com doenças crônicas graves (por exemplo: doença renal crônica resultante de diálise, cirrose Child-Pugh classe C, doença pulmonar obstrutiva crônica grave e insuficiência cardíaca grave) ou que alteram a resposta inflamatória, como aquelas que exigem o uso prolongado de imunossupressores, câncer ativo, infecção pelo vírus da imunodeficiência humana mal controlada e que recebem cuidados paliativos ou com expectativa de vida inferior a 24 horas, conforme avaliação do médico responsável.

### Procedimentos

Após a inscrição, coletaram-se amostras de sangue venoso dentro de 24 horas após a admissão na UTI. Não foi possível verificar se o sangue foi coletado antes ou depois da administração de corticosteroides. As informações sociodemográficas e clínicas foram coletadas diretamente dos pacientes ou de seus substitutos ou de prontuários médicos eletrônicos. Os níveis de BMs (cortisol, peptídeo-C, GLP-1, insulina, PYY, grelina, leptina e resistina) foram medidos usando o Metabolism/Obesity 9-Plex Human ProcartaPlex^TM^ Panel 2 (Cat. #EPX060-10824-901, Thermo Fisher Scientific, Waltham, Massachussets, Estados Unidos) e um sistema Luminex^®^ MAGPIX (Luminex Corporation, Austin, Texas, Estados Unidos). As concentrações de proteína foram calculadas usando o aplicativo *on-line* Procarta Plex Analysis Application (Thermo Fisher Scientific).

### Análise estatística

As variáveis contínuas são expressas como média e desvio-padrão (DP). Os dados categóricos são expressos como frequências e porcentagens. Os grupos de sobreviventes e não sobreviventes foram comparados usando os testes de Kruskal-Wallis e Wilcoxon-Mann-Whitney para resultados contínuos e testes de qui-quadrado para resultados categóricos; utilizou-se regressão logística binária multivariável para analisar as diferenças ajustadas entre os grupos. Além disso, utilizou-se regressão logística binária multivariável para determinar as associações entre o desfecho primário, a mortalidade hospitalar e as variáveis independentes, que foram conceitualmente divididas em fatores demográficos essenciais, incluindo idade e sexo, e fatores prognósticos clínicos (*Simplified Acute Physiology Score* [SAPS] III, Índice de Comorbidade de Charlson, escore de tomografia computadorizada de tórax [CT], *Sequential Organ Failure Assessment* [SOFA] e índice de massa corporal [IMC]), doravante denominados “preditores essenciais”, e BMs. Os últimos foram categorizados em quintis, devido às suas distribuições altamente não normais. As variáveis foram incluídas na regressão se fosse atingido um limite de p < 0,20 na análise univariada ou se houvesse plausibilidade biológica associada ao desfecho. As curvas *receiver operating characteristic* (ROC) dos preditores principais isoladamente e dos preditores principais mais os preditores de BMs foram comparadas em termos de precisão, medida usando a área sob a curva ROC (ASC ROC), bem como a sensibilidade, a especificidade, os valores preditivos positivos e negativos e as razões de verossimilhança de testes positivos e negativos. A ASC ROC foi validada de forma cruzada em cinco amostras independentes para evitar o ajuste do modelo e a avaliação da mesma amostra. Os intervalos de confiança de 95% (IC95%) com correção de viés de *bootstrap* foram usados para expressar a incerteza da ASC ROC. Utilizou-se o coeficiente de correlação de Spearman para avaliar as correlações de pares entre as variáveis numéricas. Um agrupamento hierárquico da matriz de correlação correspondente foi usado para identificar os agrupamentos de biomarcadores coexpressos. Além disso, foi realizada uma análise de agrupamento não hierárquico *K-means* para avaliar o agrupamento BMs.

Os dados foram analisados usando o IBM SPSS Statistics versão 22.0 (IBM Corp., Armonk, Nova Iorque, Estados Unidos) e o Stata versão 13.1 (StataCorp, College Station, Texas, Estados Unidos). O nível de erro tipo I foi definido como 0,05 para todas as análises estatísticas.

## RESULTADOS

### Características dos participantes

O estudo incluiu 213 indivíduos, dos quais 53 (25%) morreram durante a hospitalização; a tabela 1 apresenta uma comparação entre os que morreram e os sobreviventes. Idade avançada, IMC mais alto, necessidade de ventilação mecânica, extensão da pneumonia por COVID-19 na TC, gravidade da doença na admissão na UTI (medida pelo SAPS III), comorbidades (medida pelo Índice de Comorbidade de Charlson) e grau de disfunção orgânica (medida pelo SOFA) foram associados à mortalidade hospitalar. Após o ajuste, somente a pontuação SOFA na admissão e a pontuação SAPS III foram independentemente associadas à mortalidade hospitalar.

**Tabela 1:** Características dos participantes

	Sobrevivente (n = 160)	Não sobrevivente (n = 53)	Valor de p não ajustado	RC ajustada (IC95%)
Sexo, masculino	99 (62)	37 (70)	0,29	NA
Idade	53 ± 15	62 ± 13	< 0,001	1,02 (0,98 - 1,07)
Etnia, branca	152 (95)	48 (90)	0,24	NA
IMC	26 ± 10	29 ± 5	0,026	1,02 (0,95 - 1,09)
Tempo de início dos sintomas (dias)	10,5 ± 3,7	10,9 ± 4,2	0,67	NA
Transferido de				
Emergência	115 (72)	37 (70)		NA
Ala de COVID-19	9 (6)	4 (8)	0,87	
Outro hospital	36 (22)	12 (22)		
Necessidade de ventilação mecânica	46 (29)	37 (70)	< 0,001	NA
Extensão > 50% das lesões na TC do tórax	51 (32)	28 (53)	0,006	0,87 (0,5 - 1,5)
Índice de comorbidade de Charlson	1,62 ± 1,5	2,34 ± 1,65	0,004	0,94 (0,67 - 1,3)
*Diabetes mellitus*	14 (9)	7 (13)	0,34	NA
Uso de corticosteroides	122 (76)	44 (83)	0,30	NA
SOFA na admissão	2 [1 - 4]	4 [2 - 8]	< 0,001	1,2 (1,09 - 1,5)
Escore SAPS III	39 ± 25	58 ± 20	< 0,001	1,02 (1,004 - 1,04)

RC - razão de chances; IC95% - intervalo de confiança de 95%; NA - não se aplica; IMC - índice de massa corporal; TC - tomografia computadorizada; SOFA - *Sequential Organ Failure Assessment*; SAPS - *Simplified Acute Physiologic Score*. Resultados expressos como n (%), média ± desvio-padrão ou mediana [intervalo interquartil].

### Biomarcadores metabólicos e gravidade da doença

Na análise dos participantes de acordo com a escala de gravidade da OMS, os níveis de cortisol, resistina, leptina, insulina e grelina diferiram significativamente entre os grupos (Figura 1). Os níveis plasmáticos de cortisol e resistina foram mais altos em indivíduos ventilados mecanicamente do que naqueles com doença menos grave (Figura 1A e B). Os níveis de grelina e insulina foram maiores em indivíduos hospitalizados (categorias 5 a 7 da OMS) do que naqueles com doença leve (Figura 1C e D). Além disso, os níveis de leptina foram maiores em indivíduos com doença da categoria 5 da OMS do que em indivíduos com doença das categorias 2 - 3 e 6 ou 7 da OMS (Figura 1E). Níveis mais baixos de grelina e mais altos de cortisol foram as únicas variáveis significativamente associadas à mortalidade (Figura 2); no entanto, após o ajuste para variáveis clínicas, “níveis mais altos de cortisol” foi o único biomarcador independentemente associado à mortalidade (razão de chances [RC]: 1,003; IC95% 1,00 - 1,006; AUROC 0,61 [IC95%] 0,51 - 0,7). O IMC e os BMs medidos não foram significativamente correlacionados, exceto por uma correlação fraca com o nível de leptina (r = 0,15, p = 0,028) (Figura 3). Como o cortisol poderia ter uma associação não linear com a mortalidade, analisamos os quartis dos níveis de cortisol, mas somente os níveis mais altos de cortisol foram associados à mortalidade (Tabela 2).

**Figura 1:** Biomarcadores metabólicos e gravidade da COVID-19.

**Figura 2:** Biomarcadores metabólicos e mortalidade por COVID-19. PYY - peptídeo YY; peptídeo-1 semelhante ao glucagon.

**Figura 3:** Correlações de Spearman entre os níveis de biomarcadores metabólicos e o índice de massa corporal. PYY - peptídeo YY; GLP-1 - peptídeo 1 semelhante ao glucagon; IMC - índice de massa corporal.

**Tabela 2:** Quartis de níveis de cortisol e mortalidade

Níveis de cortisol	Sobrevivente (n = 160)	Não sobrevivente (n = 53)	Valor de p
Quartil 25	46 (82)	10 (18)	
Quartil 25 - 50	47 (84)	9 (16)	0,046
Quartil 50 - 75	34 (65)	18 (35)	
Quartil 75	33 (67)	16 (33)	

Resultados expressos como n (%).

Utilizou-se a regressão logística multivariada para investigar a capacidade dos BMs de prever a mortalidade hospitalar. A adição de BMs aos principais preditores clínicos (Tabela 3) melhorou significativamente a ASC ROC de 90,31% (IC95% 70,78 - 85,33%) para 92,65% (IC95% 86,86 - 95,74%). Também foram observadas melhorias significativas na sensibilidade (de 40 para 70%, p < 0,001), no valor preditivo negativo (de 82 para 89%, p = 0,014), na precisão (de 80 para 93%, p = 0,003) e nas razões de verossimilhança de teste positivo ou negativo (p < 0,001). A duplicação da razão de verossimilhança de teste positivo com a adição de BMs dobrou as chances de prever corretamente a mortalidade hospitalar. Dentro do intervalo de 10 a 15% de resultados de testes falso-positivos, que foi considerado aceitável em muitas das aplicações de triagem, os BMs melhoraram a previsão correta da mortalidade hospitalar em aproximadamente 10 a 30% em comparação com a ROC sem os BMs (Tabela 3 e Figura 4).

**Tabela 3:** Comparação de parâmetros de diagnóstico de dois modelos de sobrevida intra-hospitalar derivados de regressão logística multivariável e validação cruzada de cinco vezes

Nome do parâmetro	Preditores clínicos			Preditores clínicos + BMs			Diferença de parâmetro Valor de p
	Valor	Limites IC95%		Valor	Limites IC95%		
		Inferior	Superior		Inferior	Superior	
Sensibilidade	39,6	27,59	53,06	69,81	56,46	80,48	< 0,001
Especificidade	94,3	89,28	97,12	95,07	90,17	97,59	0,400
VPP	72,4	54,58	85,30	84,09	71,00	92,00	0,112
VPN	81,7	74,05	86,00	89,40	83,48	93,47	0,014
AUROC	80,4	70,78	85,33	92,65	86,86	95,74	0,003
RV+	7,03	4,77	10,36	14,16	10,46	19,17	< 0,001
RV-	0,64	0,60	0,68	0,32	0,28	0,36	< 0,001

BMs - biomarcadores metabólicos; IC95% - intervalo de confiança de 95%; VPP - valor preditivo positivo; VPN - valor preditivo negativo; ASC ROC - área sob a curva *receiver operating characteristic*; RV+ - razão de verossimilhança positiva; RV- - razão de verossimilhança negativa. Resultados expressos como %.

**Figura 4:** Comparação da curva ROC entre a capacidade das variáveis clínicas (azul) e clínicas e biomarcadores (marrom) de prever a mortalidade hospitalar em pacientes com COVID-19, conforme determinado pela regressão logística multivariada e validação cruzada de cinco vezes.

### Agrupamento baseado em biomarcadores metabólicos

Utilizou-se o coeficiente de correlação de Spearman para avaliar a correlação entre pares dos biomarcadores medidos; os agrupamentos de biomarcadores hierárquicos coexpressos são mostrados na matriz de correlação correspondente (Figura 5). O agrupamento entre insulina/resistina e leptina reforçou a associação observada entre esses biomarcadores e a gravidade da doença (Figura 1). Esse agrupamento hierárquico foi estendido para a grelina na etapa seguinte (Figura 5), e a grelina foi associada à gravidade da doença. Notavelmente, o cortisol, que estava associado à gravidade da doença e era o único biomarcador independentemente associado à mortalidade, não se agrupou com esses biomarcadores (Figura 5). No intuito de explorar ainda mais essa possível relação, foi realizada uma análise de agrupamento não hierárquica *K-means*. Usando todos os biomarcadores medidos, foram identificados dois fenótipos: o fenótipo 1, que incluiu 147 indivíduos, e o fenótipo 2, que incluiu 66 indivíduos. As características demográficas e clínicas foram semelhantes entre os dois fenótipos (Tabela 4). Os fenótipos distintos foram associados à escala de gravidade da OMS, mas não à mortalidade (Tabela 4). Resultados semelhantes foram obtidos usando o agrupamento não hierárquico *K-means* para orientar a matriz de correlação e o agrupamento hierárquico para selecionar alguns biomarcadores para determinar os fenótipos. Foi possível obter fenotipagem semelhante usando apenas os níveis de leptina e insulina.

**Figura 5:** Correlações de Spearman entre os níveis de biomarcadores metabólicos e a análise de agrupamento hierárquico. PYY - peptídeo YY; GLP-1 - peptídeo-1 semelhante ao glucagon. A magnitude de cada correlação é indicada por uma cor, sendo que a cor vermelha indica correlação positiva e a cor azul indica correlação negativa.

**Tabela 4:** Características dos agrupamentos de biomarcadores

	Agrupamento 1 (n = 147)	Agrupamento 2 (n = 66)	Valor de p
Sexo, masculino	98 (67)	38 (58)	0,20
Idade	56 ± 16	53 ± 14	0,17
IMC	27 ± 9	28 ± 9	0,23
Tempo a partir do início dos sintomas (dias)	10,7 ± 4,1	10,5 ± 3,6	0,91
Transferido de			
Emergência	108 (73)	44 (67)	0,40
Ala de COVID-19	7 (5)	6 (9)	
Outro hospital	32 (22)	16 (24)	
Extensão > 50% das lesões na TC de tórax	58 (39)	21 (32)	0,48
Índice de comorbidade de Charlson	1,9 ± 1,5	1,5 ± 1,5	0,12
*Diabetes mellitus*	17 (12)	4 (6)	0,21
Uso de corticosteroides	115 (78)	51 (77)	0,87
Escore SAPS III	42 ± 26	50 ± 23	0,13
SOFA na admissão	3,6 ± 2,9	3,0 ± 2,7	0,081
Níveis de cortisol	41 ± 63	18 ± 37	0,002
Níveis de peptídeo-C	839 ± 858	1.258 ± 1407	0,002
Níveis de GLP	310 ± 255	424 ± 255	< 0,001
Níveis de grelina	957 ± 483	1.199 ± 564	< 0,001
Níveis de insulina	10.845 ± 2642	11.882 ± 2682	0,001
Níveis de leptina	5.581 ± 3442	18.390 ± 5994	< 0,001
Níveis de PYY	188 ± 25	189 ± 18	0,627
Níveis de resistina	8.654 ± 5.338	12.229 ± 6.111	< 0,001
Escala de gravidade da OMS			
Categoria 5	53 (36)	34 (51)	0,03
Categoria 6	36 (24)	7 (11)	
Categoria 7	58 (40)	25 (38)	
Não sobrevivente	36 (24)	17 (26)	0,84

IMC - índice de massa corporal; TC - tomografia computadorizada; SAPS - *Simplified Acute Physiologic Score*; SOFA - *Sequential Organ Failure Assessment*; GLP - peptídeo semelhante ao glucagon; PYY - peptídeo YY; OMS - Organização Mundial da Saúde; NA - não se aplica. Resultados expressos como n (%) ou média ± desvio-padrão.

## DISCUSSÃO

Neste estudo multicêntrico que examinou uma grande coorte de pacientes com COVID-19, descobrimos que diferentes BMs e fenótipos de pacientes a partir de BMs foram associados à gravidade da doença, mas apenas os níveis de cortisol foram independentemente associados à mortalidade. Além disso, os BMs melhoraram a capacidade das variáveis clínicas de prever a mortalidade. Não é possível determinar se esses biomarcadores estão significativamente envolvidos na progressão da doença para formas mais graves ou se são simplesmente um epifenômeno do complexo processo patológico que ocorre durante o desenvolvimento da COVID-19.

Um achado notável deste estudo é que não encontramos correlação significativa entre o IMC e os BMs medidos, exceto os níveis de leptina. Mesmo no caso da leptina, a correlação foi fraca, apesar de sua significância estatística. Esse achado é diferente do que observado em indivíduos saudáveis,^(21)^ mas semelhante aos achados de outro estudo recente.^(14)^ Flikweert et al.^(14)^ levantaram a hipótese de que a COVID-19 pode induzir a disfunção do tecido adiposo e alterar a secreção de adipocinas, mas esse padrão foi observado em pacientes com sepse,^(22)^ sugerindo que existe uma interação complexa entre o tecido adiposo e a inflamação nessas condições; além disso, isso pode ser secundário ao IMC médio mais baixo observado em nossa coorte, em que apenas 39% (84 de 213 pacientes com IMC ≥ 30) estavam acima do peso.

O cortisol é o biomarcador mais estudado até o momento. Neste estudo, níveis mais altos de cortisol foram associados à mortalidade hospitalar e pareceram ser um marcador prognóstico independente em pacientes graves com COVID-19. Os níveis de cortisol estão relativamente bem estabelecidos na literatura como um marcador de gravidade e preditor de mortalidade em pacientes com COVID-19.^(23-27)^

Há uma escassez de estudos de coorte de grande porte sobre BMs que não o cortisol e a gravidade da COVID-19. Os receptores de grelina foram detectados no tecido pulmonar, e estudos demonstraram que a grelina tem efeitos protetores em modelos de lesão pulmonar aguda.^(28)^Neste estudo, níveis mais altos de grelina foram encontrados em sobreviventes da COVID-19. Os níveis de grelina estão inversamente correlacionados com o tempo de ventilação mecânica e permanência na UTI, e níveis séricos mais altos de grelina estão correlacionados com a sobrevida em pacientes com sepse.^(29)^ No entanto, não encontramos nenhum estudo prospectivo que determinasse o papel da grelina na previsão do prognóstico de pacientes com COVID-19.

Em pacientes com COVID-19, a modulação do sistema inflamatório pela leptina pode contribuir diretamente para a fisiopatologia da doença, pois níveis elevados de leptina estão associados a uma resposta pró-inflamatória e à gravidade da infecção em pacientes obesos e não obesos.^(30,31)^ A leptina parece ser protetora contra infecções respiratórias, e os níveis séricos de leptina são inversamente proporcionais aos biomarcadores inflamatórios em pacientes hospitalizados por pneumonia.^(32)^ Por outro lado, durante infecções virais em pacientes obesos, níveis alterados de leptina podem levar a uma resposta pró-inflamatória de citocinas e a uma resposta deficiente à infecção.^(33)^ Pacientes com COVID-19 geralmente têm níveis séricos de leptina mais altos do que controles saudáveis, mas a relação entre os níveis séricos de leptina e a gravidade da COVID-19 não é robusta.^(14,34-38)^ Nossos resultados refletem essa incerteza. Os níveis de leptina foram maiores na categoria 5 da OMS do que em todas as outras categorias, mas não foram associados à mortalidade.

Os níveis de resistina estão aumentados em pacientes com sepse e estão associados à mortalidade, independentemente do IMC em pacientes com sepse.^(39,40)^ Os níveis de resistina são maiores em pacientes com COVID-19 do que em controles saudáveis,^(41)^ e a resistina está correlacionada com a gravidade da doença e o desfecho, independentemente da presença de obesidade ou síndrome metabólica.^(14,42,43)^ Nossos resultados são consistentes com esses achados anteriores: os níveis de resistina foram maiores em pacientes com doença da categoria 7 da OMS, independentemente do IMC, mas não previram a mortalidade.

Também estudamos o peptídeo-C, o GLP-1, a insulina e o PYY, mas os níveis de insulina foram os únicos BEs que foram maiores em pacientes com formas mais graves da doença do que em pacientes com doenças de categoria 1 e 2 da OMS. Até onde sabemos, nenhum desses marcadores foi estudado como biomarcadores prognósticos para a COVID-19. Como observado na sepse, a COVID-19 pode induzir o estresse hipermetabólico, que está associado à hiperglicemia e à resistência à insulina.^(44)^ O hiperinsulinismo é uma resposta de adaptação à liberação excessiva de hormônios contrarreguladores, e isso poderia explicar parcialmente nossos resultados.

Independentemente dos parâmetros clínicos, podem-se agrupar os pacientes com COVID-19 em dois fenótipos associados à gravidade da doença. Essa abordagem pode ser útil para oferecer um atendimento mais personalizado. Em outro estudo, cinco fenótipos de pacientes hospitalizados com COVID-19 foram identificados usando creatinina, albumina, PCR, contagem de leucócitos e características clínicas.^(45)^ Pacientes com um fenótipo caracterizado por insuficiência renal, hipoalbuminemia, anemia, linfopenia e níveis elevados de PCR tiveram a maior probabilidade de transferência para a UTI ou mortalidade hospitalar. Três subfenótipos, a saber, histórico de hipertensão crônica, febre, sintomas respiratórios e não respiratórios e idade, foram associados à deterioração clínica.^(46)^ Além disso, alguns fenótipos foram associados a melhores resultados após a introdução da terapia com dexametasona para o tratamento da COVID-19, reforçando a ideia de que a fenotipagem de pacientes pode ter um impacto no prognóstico e na estratificação do tratamento.^(47)^ Até onde sabemos, este estudo é o primeiro a fornecer fenótipos discriminativos com base apenas nos níveis de BEs.

Poucos estudos prospectivos incluíram um grande número de pacientes de diferentes centros, o que é um dos principais pontos fortes deste estudo. Além disso, este estudo é o primeiro a mostrar que os BMs podem melhorar a precisão prognóstica dos parâmetros clínicos e ser usados para fenotipar pacientes independentemente dos parâmetros clínicos. No entanto, este estudo também tem várias limitações. Primeiro, os BMs foram analisados em um único momento após a admissão hospitalar, o que levou a uma falta de informações sobre as modificações metabólicas ao longo do tempo e sua associação com a progressão da doença. Esse é um dos grandes desafios do uso de biomarcadores e da fenotipagem em doenças complexas, como a COVID-19. Acreditamos que essa limitação pode ser atenuada pelo fato de que os biomarcadores aumentam o valor prognóstico de parâmetros clínicos relevantes. Além disso, como o tempo decorrido entre o início dos sintomas da COVID-19 autorrelatados e a inclusão no estudo foi semelhante entre os grupos, isso poderia reduzir o possível viés temporal relacionado a uma única medição de ponto de tempo dos biomarcadores. Em segundo lugar, algumas adipocinas relevantes, como a adiponectina e a visfatina, não foram medidas, impedindo uma avaliação abrangente do papel dos BMs em pacientes com COVID-19. Em terceiro lugar, o controle glicêmico durante a UTI poderia afetar a mortalidade e ser impactado pelo uso de dexametasona, mas, infelizmente, não temos informações sobre esses parâmetros (como a glicemia capilar mediana e a hemoglobina glicosilada), o que deve ser observado como uma limitação do nosso estudo. No entanto, alguns estudos não associaram o controle glicêmico com a gravidade da doença, mesmo em pacientes diabéticos que receberam dexametasona.^(48)^Em quinto lugar, os fenótipos dos BMs também poderiam afetar os resultados de longo prazo, uma vez que a atrofia muscular persistente, o novo início do diabetes e outros resultados metabólicos estão associados à COVID-19,^(49-51)^e são necessários mais estudos para avaliar o papel dos BMs na previsão dos resultados de longo prazo dos pacientes com COVID-19. Em sexto lugar, só temos informações sobre o uso de corticosteroides, não sobre a dose do medicamento administrado; portanto, embora o número de pacientes que usaram corticosteroides tenha sido semelhante entre sobreviventes e não sobreviventes, é possível que haja alguns fatores de confusão, principalmente relacionados à dose usada. Entretanto, o protocolo institucional sugeriu o uso de dexametasona 6 mg, o que poderia atenuar parcialmente esse viés. Em sétimo lugar, durante a análise dos dados, realizamos comparações múltiplas, e isso traz intrinsecamente um possível viés, que deve ser levado em conta ao ler nossos resultados.

## CONCLUSÃO

O cortisol foi o único biomarcador individual independentemente associado à mortalidade; entretanto, os biomarcadores metabólicos melhoraram a previsão de mortalidade quando adicionados aos parâmetros clínicos. Os fenótipos dos biomarcadores metabólicos foram distribuídos de forma diferenciada de acordo com a gravidade da COVID-19, mas não foram associados à mortalidade. Em conjunto, esses resultados sugerem que os BMs podem desempenhar um papel na progressão da COVID-19 e podem refletir um desequilíbrio em diferentes vias afetadas pela infecção por SARS-CoV-2.
